# The Rush for the Rare: Reptiles and Amphibians in the European Pet Trade

**DOI:** 10.3390/ani10112085

**Published:** 2020-11-10

**Authors:** Sandra Altherr, Katharina Lameter

**Affiliations:** Pro Wildlife, Engelhardstrasse 10, 81369 Munich, Germany; katharina.lameter@prowildlife.de

**Keywords:** biodiversity, exotic pet trade, CITES, reptiles, amphibians, conservation

## Abstract

**Simple Summary:**

The exotic pet trade has been identified as a main threat to many reptile and amphibian species, especially for optically attractive species or those with special biological features. The international exotic pet trade is largely unregulated and in large parts still heavily depends on wild-caught specimens. Rarity sells, and species that are new to science or new on the pet market are highly sought-after and may fetch record prices. The European Union is a main hub and destination for both legally and illegally sourced wildlife. In the German town Hamm, one of the largest reptile trade shows in the world takes place four times a year, attracting traders and clients from across Europe and beyond. Based on ten case studies, our article illustrates marketing mechanisms and trade dynamics for reptiles and amphibians, which have only recently been described by science. The paper also highlights the problems of insufficient international legislation to prevent over-exploitation of such species or even those which are nationally protected in their country of origin, and presents solutions.

**Abstract:**

Direct exploitation is one of the five main reasons for the loss of biodiversity, and collections for the international pet trade are an ongoing threat for many reptiles and amphibians. The European Union and in particular Germany have a central role as a hub and destination for exotic pets from all over the world. Rare species of reptiles and amphibians especially are in the focus of collectors. Rarity on the market may be either caused by rarity of a species in the wild or by a limited availability for sale, e.g., due to national protection measures in the range state or remote localities. The present study identified 43 species that are not listed by the Convention on International Trade in Endangered Species of Wild Fauna and Flora (CITES) and were only recently described, but have already entered the European pet trade. Ten of these species were selected as case studies, representing species from different geographic regions and illustrating the marketing mechanisms. Many such species that are new to science are neither assessed by the International Union for Conservation of Nature (IUCN) Red List of Threatened Species nor are they covered by international legislation, even though in several countries, where such internationally sought-after species are caught, national protection measures are in place. This paper analyses the challenges and opportunities for the protection of potentially threatened and newly described reptile and amphibian species against over-exploitation for the pet trade.

## 1. Introduction

Direct exploitation is one of the five major threats to biodiversity [1,2]. While this includes timber extraction, global fisheries, and capture of terrestrial species for consumption, trade in exotic pets is a threat to many, often particularly rare, species which can fetch high market prices [3,4,5,6,7]. Such species, and those that have been newly described by science, are often of special interest to collectors [8,9,10,11]. Population data of recently described species are naturally scarce and thus they are neither evaluated by the IUCN Red List of Threatened Species nor are they subject to international trade restrictions [4,5,12]. The inclusion of a species in Appendix I or II of the Convention on International Trade in Endangered Species of Wild Fauna and Flora (CITES), which would prohibit or regulate international trade, is often hampered by a lack of data, economic interests and the fact that Conferences of the Parties only take place every three years [12,13,14].

Furthermore, there is increasing evidence for a systematic trade in species that are only protected in their countries of origin [15,16,17,18]. Absence of a listing in the appendices of CITES or the annexes of the EU Wildlife Trade Regulation 338/97 basically means that animals can be openly and legally imported, sold and kept in European Union Member States [4,5,18,19,20]. This substantial legal gap fuels a dubious business with extremely high profits without the risk of legal consequences [21,22]. Thus far, the USA is the only country that prohibits the trade in nationally protected species through its Lacey Act, 16 U.S.C. §§ 3371–3378, which says: “It is unlawful for any person … (2) to import, export, transport, sell, receive, acquire, or purchase in interstate or foreign commerce—(A) any fish or wildlife taken, possessed, transported, or sold in violation of any law or regulation of any State or in violation of any foreign law;…”.

The EU plays a central role as a hub and destination for both legally and illegally traded wildlife [4,5,19,23,24,25,26]. Within the EU, Germany in particular plays a leading role in the exotic pet trade and German keepers have a long tradition of keeping reptiles and amphibians [27,28]. One of the world’s largest reptile trade shows, the Terraristika, takes place four times a year in the town of Hamm (northern Germany) and attracts traders and potential clients not only from all over Europe, but from across the world [4,15,29]. Moreover, Germany is by far the largest importer of live reptiles within the EU [30]. In 2016, the European Union passed its Action Plan against wildlife trafficking, which, among others, has the target, to “reduce or ban unsustainable imports into the EU of endangered species by proposing their listing in CITES Appendices (e.g., rare reptile species)” [31]. As part of Germany’s national Action Plan the German Federal Ministry for the Environment and the Federal Agency for Nature Conservation commissioned a study, which identified more than 2000 species of reptiles, amphibians, and non-domesticated mammals in the German pet trade within September 2017 and September 2018 [32]. The resulting database was a main source for the present paper.

## 2. Materials and Methods

To analyze the potential threat that the pet trade presents to newly described and potentially threatened species, this paper focuses on reptile and amphibian species that were not protected under CITES at the time the data were recorded, assuming that these species are under particular threat from unregulated trade. Two sets of data were used for the present study.

The first set of data were derived from the study “Strategies for reducing demand for reptiles, amphibians and small mammals kept as pets”, which we compiled for a project that was commissioned by the German Federal Agency for Nature Conservation (BfN) and the German Federal Environmental Ministry (BMU) on reptile, amphibian and non-domesticated mammal species [32]. The resulting database contains all species, which had been offered for sale over a twelve-month period (September 2017 to September 2018) on five major online platforms, five Facebook sales groups and five cross-regional reptile trade shows. Recording of offers was limited to traders from Germany and offers for trade shows in Germany. In order to exclude invalid taxa, the scientific validity of species names were verified using the taxonomic Reptile Database [33], the Amphibian Database by the University of California [34] and the Mammal database of the National Museum of Natural History Smithsonian Institution [35]. Statements by traders highlighting the rarity of a species—either in the wild or on the international market—were also noted.

For each species, we supplemented information on a range states, IUCN Red List status, population trends, year of scientific description, protection status under CITES and within the European Union (via EU Wildlife Trade Regulation 338/97) using AmphibiaWeb [34], the Reptile Database [33], the IUCN Red List 2020 [36], the WISIA database by the German Government on legal status of species [37] as well as market prices (own records). Furthermore, for non-CITES listed species information on potential national protection status in the countries of origin was added into the database. For further analysis, only non-CITES reptile and amphibian species described within the period 2008–2017 were selected, resulting in dataset 1 (see Table A1).

A second dataset was compiled in order to provide the most up-to-date data for the present paper and to consider species that were only described after the closure of dataset 1 in 2018. We thus screened the online platform terraristik.com, which had been identified in the abovementioned research project as the most relevant trade channel [32], for the keywords “rare”, “first time”, and “new species”. For recorded species, a literature survey was conducted at Google Scholar to identify the year of first description, recent publications providing new records in the wild, as well as data on conservation status and trade. Only species in the European pet trade that were not yet covered in dataset 1 and described only since 2008 were included in dataset 2 (see Table A1).

Finally, 10 of the 43 species of datasets 1 and 2, which were offered for sale shortly after their description and some of them being nationally protected, were selected as case studies, choosing examples from different taxonomic groups (snakes, lizards, and amphibians), as well as different countries and geographic regions (see Table 1).

## 3. Results

### 3.1. Number of Recorded Species

Within the 12-month period between September 2017 and September 2018, 1532 valid reptile species and 352 valid amphibian species had been recorded in the German pet trade [32]. Within that investigation period, a total of 46 reptile and amphibian species were identified in the European pet trade, which had only been scientifically described within the period of 2008–2017; 38 of which were not protected by CITES at that time (dataset 1, see Table A1).

**Figure 1:**
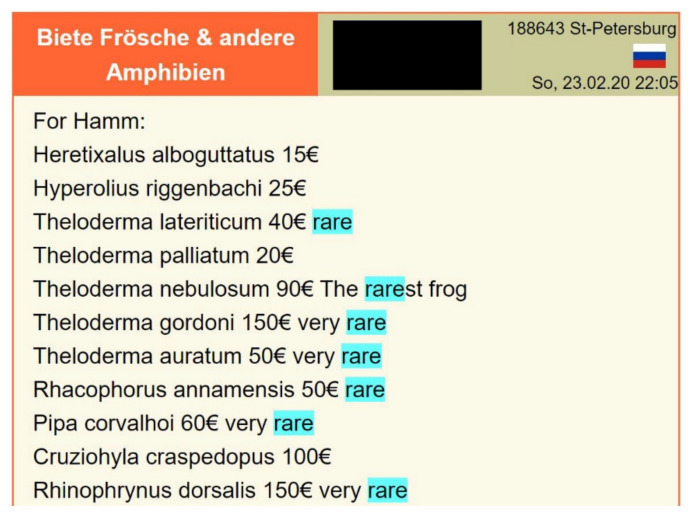
Online advertisement at www.terraristik.com by a Russian trader as of February 2020 (viewed 28 August 2020), advertising different amphibian species and highlighting the rarity of some. To be sold in “Hamm”, the Terraristika reptile trade show in Germany. Screenshot from terraristik.com of 25 May 2020.

For the present study, in a second online screening in July and August 2020, eleven additional reptile and amphibian species were recorded, which were offered as “first time” in trade, “rare” or “new” species (see example in Figure 1). Three of those species were already described prior to 2008 and were also recorded during our first study period: Annam flying frog (*Rhacophorus annamensis*), Mexican burrowing toad (*Rhinophrynus dorsalis*) and Gordon’s Bug-Eyed Frog (*Theloderma gordoni*). While the Carvalho’s Surinam toad (*Pipa corvalhoi*) was newly recorded, it had already been described prior to 2008; the cloaked moss frog (*Theloderma palliatum*) was only described in 2011, but was already covered by our database 1. While the fringed leaf frog (*Cruziohyla craspedopus*) was already described in 1957, new records were published in 2017 and 2019, followed by offers in trade (see Figure 1). Those six species were not included in our dataset 2.

Accordingly, five of the eleven “rare” or “new” species on sale were newly described species: One lizard species of “*Potamites* sp. Nov.” is not yet scientifically described, which hinders further analysis. The brick-red bug-eyed frog (*Theloderma lateriticum*) was described in 2009 and, to our knowledge, was not recorded in trade before 2019. Sylvia’s tree frog (*Cruziohyla sylviae*) and the golden bug-eyed frog (*Theloderma auratum*) were both scientifically described for the first time in 2018 and traced by the authors for the first time in the European pet trade in 2019. Finally, the misty moss frog (*Theloderma nebulosum*) was described in 2011 and has been recorded in the pet trade at least since 2019 (see Table 1). The last three species were selected for further analysis and portrayed in Table 1. In total, datasets 1 and 2 resulted in 43 species recorded in the trade, and were only recently described (see Table A1).

### 3.2. Limited Distribution

Ten species were summarized as case studies in Table 1, representing new species from twelve different countries and three different taxonomic groups: The lizards Persian striped skink (*Eumeces persicus*), Hispaniolan dune curlytail (*Leiocephalus sixtoi*), Pethiyagoda’s crestless lizard (*Calotes pethiyagodai*), Lauhachinda’s cave gecko (*Gekko lauhachindai*) and the occidental chameleon gecko (*Eurydactylodes occidentalis*); the venomous snakes Emerald horned pitviper (*Ophryacus smaragdinus*) and Kimberley death adder (*Acanthophis cryptamydros*), and finally, the amphibians Sylvia’s tree frog (*Cruziohyla sylviae*), golden bug-eyed frog (*Theloderma auratum*) and the misty moss frog (*Theloderma nebulosum*). Except *Cruziohyla sylviae*, with its four confirmed range states, all these species are endemic, i.e., restricted to one single range state. Some species are limited to very small Areas of Occupancy (AOO), such as *Gekko lauhachindai* with an AOO of less than 10 km^2^ [57], *Eurydactylodes occidentalis* with an AOO of only 2.5 km^2^ [58], and *Calotes pethiyagodai* with an AOO of less than 25 km^2^ [51]. For *T. nebulosum* no AOO has been defined but its Extent of Occurrence (EOO) is considered to be only 940 km^2^ [54]. For some species, such as *Eumeces persicus*, *Eurydactylodes occidentalis* and *Theloderma auratum*, only very few localities are known. Other species, such as *Leiocephalus sixtoi*, *Calotes pethiyagodai*, *Gekko lauhachindai* and *Theloderma nebulosum* are known only from a single locality (details are given in Table 1).

### 3.3. New Species in Trade

Some species appear in trade almost immediately after their scientific description (within a few months). For all ten case studies the original scientific description published precise GPS data of the type localities (for holotype and/or paratypes), based on which traders can easily organize collection from the wild. For the portrayed case studies, the shortest recorded period between scientific publication and sale in Europe was only three months in the case of *Eumeces persicus*, while in most cases the period from description to first marketing takes one to three years [32] (see Table 1).

### 3.4. Marketing Mechanisms for Rare and New Species in Trade

We also found that scientific publications of new species (or new records of already known species) are often accompanied by media articles or hobbyist magazines, highlighting special features, such as “the new snake is among world’s most venomous” (*Acanthophis cryptamydros*), “very bright colors” (*Calotes pethiyagodai*), or “one of the world’s most spectacular frogs” (*Cruziohyla sylviae*). Traders can achieve higher prices for rare species and therefore underline the rarity (see Figure 1). Hence, advertisements often use terms such as “rare opportunity”, “first ever offered” or “only a few collections have them in all world”. *Theloderma nebulosum* for example, was praised as “the rarest frog”.

In particular, those reptiles and amphibians, which are new in trade and those with special biological features such as bright colors, attractive patterns, venomousness, viviparity or unique taxonomic status, may also easily reach 1000 EURO and more per animal, such as *Eurydactylodes occidentalis* (market price in 2017 about 2000 EURO per pair). *Ophryacus smaragdinus* was offered as “one of the rarest venomous snakes and a crowned jewel of any collection” in 2017, prices of 1700 EURO per animal were demanded (see Table 1). Such record prices are requested for the first animals made commercially available, whereas prices usually drop when more specimens enter the market [32].

### 3.5. Conservation and Protection Status

Several newly described species appeared in trade before they were assessed for the IUCN Red List (e.g., *Cruziohyla sylviae*, *Eumeces persicus*, *Ophryacus smaragdinus*, *Theloderma auratum*). Accordingly, information on, e.g., population status or trends, is lacking, which would be a precondition to assess the impact of collections. For other species, such as *Calotes pethiyagodai*, a classification in the global IUCN Red List assessment has not yet been conducted, while the conservation action plan of the sole range state Sri Lanka recommends the species as Endangered [52]. However, national recommendations and classifications in national Red Lists are often less communicated by authorities, and therefore less known to stakeholders from abroad.

For some of the case studies in Table 1 the assessment in the IUCN Red List stated that “no utilization of the species is known”, which is the case for, e.g., *Acanthophis cryptamydros* [49] and *Gekko lauhachindai* [57]. Hence, in such cases data on international trade are not yet included, which may lead to a less alarming classification in the IUCN Red List and eventually even reduce potential CITES initiatives; however, content heavily depends on the assessors and on regular updates.

Many range states of potentially threatened species have taken national protection measures, e.g., prohibiting any commercial exports or only allowing exports under special permits, exemplified by Sri Lanka, Costa Rica and Australia that do not allow any commercial export of native reptiles and amphibians (see Table 2). Accordingly, offers of only recently described and nationally protected species within the European pet market indicate illegal captures and exports, such as for *Calotes pethiyagodai*, *Acanthophis cryptamydros* or *Cruziohyla sylviae*.

In other countries, for example Mexico or Iran, exports require a permit by national authorities (see Table 2), which are only issued for a few species [19]. Other countries in principle allow the commercial export of species under certain circumstances, but the species in trade is restricted to protected areas or selected species that are protected and, accordingly, exports for wild-caught animals of those species are not allowed. Countries with strict legislation sometimes are not aware of the international trade in their native species.

## 4. Discussion

The exotic pet trade presents a serious threat to wild populations of reptiles and amphibians [2,55,60,61,62,63,64]. The European Union is a main hub and destination for potentially threatened species [4,5,8,16,19,20,23]. Especially species with striking colors or patterns, a special biology, such as viviparity, venomousness, or a special taxonomic status are highly sought-after [4,5,9,25,29,32]. These attributes, driving demand, also apply to several species portrayed in Table 1, such as *Acanthophis cryptamydros*, *Ophryacus smaragdinus*, *Calotes pethiyagodai*, *Cruziohyla sylviae*, and *Theloderma auratum*.

The limited availability at the international market is a second factor, which makes species, such as *Calotes pethiyagodai*, *Eurydactylodes occidentalis*, *Gekko lauhachindai*, *Ophryacus smaragdinus*, or *Theloderma nebulosum*, attractive for special clients, who are willing to pay high prices for such animals [15,65,66,67,68]. Such limited availability in international trade may either be due to rarity in the wild (e.g., restricted-range species, populations with a low density, species displaying a cryptic life history) or rarity on the market [4,5,69], e.g., due to remoteness of the distribution area of a species, lack of field data and information on localities, or national export bans in the range state (e.g., Australia, Costa Rica, or Sri Lanka) [15,17,20,23,70]. Especially species with small distribution range, such as *Gekko lauhachindai*, *Theloderma nebulosum*, *Calotes pethiyagodai*, or *Eurydactylodes occidentalis* are extraordinarily susceptible to over-exploitation [42,43] and recent trade records are highly alarming. Furthermore, we acknowledge that our trade records provide a point in time, since when a species was at least commercially available in the European Union is not necessarily the first appearance of the species in trade, e.g., other trade channels may have been used or offers may have been removed prior to our surveys.

For all of the case studies presented in Table 1, scientific descriptions of newly discovered species provided detailed GPS-data of type localities (holotype and/or paratypes), which facilitated access for collectors. The problem of divulging natural sites for rare species is not limited to recently described forms. Species that have long been known to science and that have evaded commercial collection also become targeted and appear in trade due to the publication of new localities in scientific papers. This issue has been observed, for example, regarding *Cruziohyla craspedopus* (described in 1957) and *Lanthanotus borneensis* (described in 1877), after new localities were recently published [20,64,71,72]. Thus, in such cases, access to localities with new or rare species may be facilitated for collectors, and legislation and enforcement in these areas may be weaker due to previous under-ascertainment of population states [17,20]. Reasonably, an increasing number of field herpetologists and conservation scientists caution against the publication of precise localities when reporting newly described species or potentially threatened species, in order to protect them from over-exploitation [11,64,70,73]. However, while scientists must understandably document their work precisely, important cautious consideration should be urged where possible revealing sufficient detail to attract unscrupulous collectors, who may learn of species localities via general sources such as media interviews or hobbyist magazines. In addition, scientific journals should pass a policy not to publish detailed data regarding the locations of relevant vulnerable species.

Seventy-five percent of reptile species and more than 80 percent of amphibian species in the exotic pet trade are not listed in the CITES Appendices [3,21,32,60]. While a CITES listing does not automatically mean that a species is protected, it does, if properly implemented, regulate the international trade. Apart from CITES, there is no international mechanism that can prevent over-exploitation for the international trade [12,14,21]. While the USA is at least recording all wildlife imports (and exports) at the species level via its LEMIS database, the European Union does not record such species-specific import and export data, apart from a small number of species that are listed in the EU Annex D. While Annex D aims to record import data, it does not at all regulate imports. Furthermore, it is only used by some Member States and therefore only providing incomplete data.

While the protection of species under CITES is a helpful tool to regulate (Appendix II) or even prohibit (Appendix I), the international commercial trade in threatened species, it is a time consuming and sometimes highly political procedure, with the Conference of the Parties (CoP) only taking place every three years. Initiatives for proposals are often hindered by a lack of precise trade data and information on wild populations, as well as by economic interests [4,5,12,13,74]. Accordingly, while CITES maybe currently the best available tool to regulate international trade in wild species, this framework, which only covers a small portion of species in trade, is reactive, does follow a precautionary principle, and leaves trade in most reptile and amphibian species unmonitored and unregulated [3,14,74].

The fact that the European Union is a main hub and destination for rare species is also reflected by the case studies given in Table 1, where species such as *Calotes pethiyagodai* or *Eumeces persicus* were first available at the European pet market and were later offered by traders in the USA or Canada. Such market dynamics have also been described for other species, such as for several reptiles endemic to Sri Lanka and for the Borneo earless monitor lizard (*Lanthanotus borneensis*). It has therefore been supposed that the European market may be used as a channel for US traders and clients to circumvent the US Lacey Act [4,16]—a law that makes import, trade and possession of specimens, which were caught and exported in violation of the legislation in the country of origin, an offense in the USA.

Many of the species, which were only recently described and are already in trade, are native to countries with national legislation protecting fauna against commercial exploitation. Australia, Costa Rica, Mexico, New Caledonia, Sri Lanka and Vietnam are among these countries and have national protection measures for their native fauna, but are known targets of wildlife traffickers [4,16,19,75]. In its Wildlife Crime Report 2020 the United Nations Office on Drugs and Crime (UNODC) stated:

“[…] while global research on wildlife crime has mainly focused on internationally protected species, little comparative analysis is available on wildlife crime affecting nationally protected species, including illegal domestic trade. With criminals taking advantage of any loophole, there is a need to better understand the trafficking of non-CITES listed species within and across borders to support law enforcement and criminal justice practitioners to define national and international tools that can protect the biodiversity of each country from criminal threats” [76].

Several experts, conservationists and political bodies, including the former CITES General Secretary, the European Parliament, and the European Union Action to Fight Environmental Crime (EFFACE) Research Project, have called on the European Union as a main hub and destination to pass legislation following the model of the US Lacey Act, in order to combat wildlife trafficking in nationally protected species [15,16,32,60,77,78,79,80].

## 5. Conclusions

Rarity sells and several species new to science are highly sought-after in the international pet trade. Despite warnings by an increasing number of field herpetologists and conservations to refrain from publishing the precise locations of new species (and equally for new records of known species), such data are still provided in many recent scientific descriptions. Those data can be used to systematically trace and collect animals from the wild, as was noted for all of our case studies. It is therefore imperative that the scientific community, including journals, reposition themselves in order not to publish locality details for species discoveries.

Furthermore, in the absence of a US-Lacey-Act-like, regulation of any non-CITES reptile or amphibian, taken in violation of legislation in their country of origin, can be legally imported and marketed in the European Union [15,16,17,77]. As long as importing countries condone the marketing of illegally sourced species, the exotic pet trade remains a driver for the loss of biodiversity. The authors therefore recommend that the European Union passes legislation comparable to the Lacey Act in the USA. This would not only support range states in their conservation efforts but would also help to combat the trade of many potentially threatened species.

Finally, given that trade in the vast majority of reptile and amphibian species is not regulated because they are not included in the CITES Appendices and because of the slow process of listing species, a reversal of the burden of proof for international wildlife trade should be considered. For example, Marshall et al. (2020) recommend only allowing trade in wildlife after it has been proven that collections are not detrimental to wild populations [3]. The Treaty on the Functioning of the European Union in Article 191 already requires EU policy to be based on precautionary principles and on the principles that preventive action should be taken.

## Figures and Tables

**Table 1:** Case studies of recently described reptile and amphibian species. Sorted by “Year of description”. IUCN Red List classifications: CR = Critically Endangered; EN = Endangered, VU = Vulnerable, DD = Data Deficient, NE = Not Evaluated. AOO = Area of Occupancy. Species of dataset 1 are marked with *, species of dataset 2 with **.

Species	Distribution	Year of Description	Recorded in Trade	IUCN Red List	Information on Conservation and Trade
Cruziohyla sylviae ** Sylvia’s tree frog	Costa Rica, Honduras, Nicaragua, Panama	2018	2019	NE	First description named detailed GPS data in Costa Rica [38]. Accompanying news articles praised species as “one of the world’s most spectacular frogs” [39]. Offered in European pet trade since at least July 2019 [15]. On sale are also animals with reference to localities in Costa Rica, while Costa Rica does not allow commercial exports of its wildlife.
Theloderma auratum ** Golden bug-eyed frog	Vietnam	2018	2019	NE	Only known from five localities, of which at least two are in protected areas, GPS data provided [40]. Recommended as DD for IUCN Red List [40]. Frogs of the genus *Theloderma* spp. have become popular in international pet trade [32]. *T. auratum* since 2019 offered as “very rare” (see Figure 1).
Eumeces persicus * Persian striped skink	Iran (only Tehran Province)	2017	2017	NE	Only known from two localities, GPS data provided [41]. Small distribution range makes lizard species susceptible to over-exploitation [42,43]. Almost no information on this species available. First record in pet trade in Germany only three months after scientific description [32].
Leiocephalus sixtoi * Hispaniolan dune curlytail	Dominican Republic	2016	2017	NE	Only known from vicinity of type locality: coastal dunes of Bahia de las Calderas [44]. Small distribution range makes lizard species susceptible to over-exploitation [42,43]. GPS data in scientific description [45] At least since 2017 in European pet trade, offered by a German trader [32].
Ophryacus smaragdinus * Emerald horned pitviper	Mexico	2015	2017	NE	Restricted to humid windward slopes of Sierra Madre Oriental in eastern Mexico; GPS data provided [46]. Extremely vulnerable to any threat in its limited range, candidate for immediate protection, illegal collection as a threat [46], no commercial exports permitted. Praised as “one of the rarest venomous snakes and a crowned jewel of any collection”, price in 2017 ~1700 EURO [32]. Not yet assessed in IUCN Red List, but in Mexico assessed as “high vulnerability species” and recommended for VU [47].
Acanthophis cryptamydros * Kimberley death adder	Australia	2015	2018	VU	Restricted to the Kimberley region, Western Australia; GPS data provided [48]. Decreasing population [49]. “new snake is among world’s most venomous” [50]. IUCN Red List: “not known from the pet trade at present” [49]. Despite strict export ban in sole range state species recorded in European pet trade in 2018 [32].
Calotes pethiyagodai * Pethiyagoda’s crestless lizard	Sri Lanka	2014	2016	(EN) ^1^	Only known from Knuckles Massif, AOO less than 25 km^2^, GPS data provided [51]. Sri Lanka does not allow any legal exports for commercial trade; but pet trade considered as threat for this species [52]. Small distribution range makes lizard species susceptible to over-exploitation [42,43]. Offered in Europe for the first time in Nov 2016; ~1000 USD per pair, praised for their bright colours [53], more offers recorded in 2017 and 2018 [32]. CITES proposal at CoP18 was withdrawn.
Theloderma nebulosum * Misty moss frog	Viet Nam	2011	2019	EN	EOO only 940 km^2^, decreasing population [54]. Only known location is in Ngoc Linh Nature Reserve, GPS data provided [55]. Scientists recorded only one adult female and eight larvae of this species in the wild [54]. Nevertheless noted in European pet trade at least since 2019, offered as “the rarest frog” (see Figure 1).
Gekko lauhachindai * Lauhachinda’s cave gecko	Thailand	2010	2018	CR	First description provided detailed GPS data [56]. AOO < 10 km^2^, occurring at a single location, very small and decreasing population [57]. Small distribution range makes lizard species susceptible to over-exploitation [42,43] While IUCN assessment states “no known use of or trade in this species” [57], repeatedly documented in Europe at least since 2018 [32].
Eurydactylodes occidentalis * Occidental chameleon gecko	New Caledonia	2009	2017	CR	Restricted to two locations, AOO calculated at 2.5 km^2^, severely fragmented habitat, decreasing population [58]. Small distribution range makes lizard species susceptible to over-exploitation [42,43]. First description provided detailed GPS data [59]. Protected in Province Nord and Province Sud, “the distinctive chameleon-like appearance of this species and its diurnal activity make it a potential target for illegal collection and trafficking” [58] In the international pet trade since at least 2017, prices up to 2300 USD per pair, in Europe in 2019 sold for 1000 EURO per specimen [32].

^1^ (IUCN Red List Classification in brackets) = so far only recommended.

**Table 2:** National legislation in different range states.

Country	National Legislation for Native Wildlife
**Australia**	The Environment Protection and Biodiversity Conservation Act 1999 prohibits export of live native Australian mammals, birds, reptiles and amphibians for commercial purposes. Native wildlife was previously protected by the Wildlife Protection (Regulation of Exports and Imports) Act 1982
**Costa Rica**	The Wildlife Conservation Law No.7317 of 1992 (Ley de Conservación de la Vida Silvestre) and Regulation 40548 of 2017 prohibits the removal of wild animals without special authorization from the government. Exports of wildlife for scientific purposes require a permit, while exports for commercial purposes are prohibited.
**Iran**	In accordance with the Environmental Protection and Enhancement Act (1974) and the Executive ByLaw on the Game and Fish Law (1967) any hunting, killing or catching of all wild mammals, birds and reptiles as well as fishing, killing or catching aquatic animals is prohibited. The same applies to any export of live wild animals without a license or approval from the Department of Environment.
**Mexico**	The “NORMA Oficial Mexicana NOM-059” identifies and lists threatened native species and populations, (SEMARNAT 2010). In accordance with Article 420 of the Código Penal (Criminal Code), capture of and commercial activity with wild animals and plants, which are endemic, in danger of extinction, threatened, rare or subject to special protection, is prohibited without proper permit. Legal commercial exports for species covered by NOM-059 are exceptional and limited to few species and specimens
**Sri Lanka**	In accordance with Section 30 of the Fauna and Flora Protection Ordinance (FFPO) of Sri Lanka in 1993, all reptiles and amphibians are protected, and thus must not be collected, including outside of protected areas. Section 40 of the FFPO completely prohibits the export of any reptile from Sri Lanka, including eggs or parts, without a permit from the Director General of the Department of Wildlife Conservation. Such exceptional permits are only possible for the promotion of scientific knowledge and research.

## Appendix A

**Table A1:** Species only described since 2008 and recorded in the European pet trade. Ordered by taxa and alphabet. Species of dataset 1 are marked with *, species of dataset 2 with **. Underlined species were selected as case studies. CR = Critically Endangered, DD = Data Deficient, EN = Endangered, LC = Least Concern, NE = Not Evaluated, NT = Near Threatened, VU = Vulnerable.

Species	Common Name	Year of Description	CITES App.	IUCN Red List
**LIZARDS**				
***Acanthosaura bintangensis ****	Bukit Larut mountain horned agamid	2009	-	DD
***Afroedura maripi ****	Marip flat gecko	2014	-	LC
***Anolis eladioi ****	Baoruco green anole	2016	-	NE
***Calotes bachae ****	Vietnamese blue crested lizard	2013	-	LC
***Calotes pethiyagodai ****	Pethiyagoda’s crestless lizard	2014	-	NE[EN] ^1^
***Cyrtodactylus doisuthep ****	Doi Suthep bent-toed gecko	2014	-	LC
***Cyrtodactylus lekaguli ****	Tuk-kai Boonsong bent-toed gecko	2012	-	LC
***Egernia cygnitos ****	Red pygmy spiny-tailed skink	2011	-	LC
***Egernia eos ****	Central pygmy spiny-tailed skink	2011	-	LC
***Egernia epsisolus ****	Eastern Pilbara spiny tailed skink	2011	-	LC
***Eumeces persicus*** *******	Persian striped skink	2017	-	NE
***Eurydactylodes occidentalis*** *******	Occidental chameleon gecko	2009	-	CR
***Flexiseps meva ****	---	2011	-	NE
***Gekko lauhachindai*** *******	Lauhachinda’s cave gecko	2010	-	CR ^2^
***Gekko shibatai ****	Takara gecko	2008	-	VU
***Gekko vertebralis ****	---	2008	-	LC
***Goniurosaurus catbaensis ****	Cat-Ba tiger gecko	2008	II (2019)	EN
***Goniurosaurus huuliensis ****	Huulien tiger gecko	2008	II (2019)	CR
***Goniurosaurus liboensis ****	Libo leopard gecko	2013	II (2019)	EN ^2^
***Hemidactylus alkiyumii ****	Al Kiyumi’s leaf-toed gecko	2012	-	NE
***Hemidactylus imbricatus ****	Carrot-tail viper gecko	2008	-	LC
***Hemidactylus kyaboboensis ****	Kyabobo forest gecko	2014	-	NE
***Diploderma yulongensis ****	Enigmatic mountain dragon	2012	-	NE
***Leiocephalus sixtoi*** *******	Hispaniolan dune curlytail	2016	-	NE
***Oedura fimbria ****	Western marbled velvet gecko	2016	-	LC
***Paroedura stellata ****	---	2011	-	NE
***Phymaturus delheyi ****	---	2011	-	LC
***Polamites sp. nov. *****	---	-	-	NE
***Pseudocalotes ziegleri ****	Ziegler’s tree lizard	2010	-	DD
***Ptyodactylus dhofarensis ****	Dhofar fan-footed gecko	2013	-	NE
***Saltuarius kateae ****	Kate’s leaf-tailed gecko	2008	-	LC
***Thecadactylus oskrobapreinorum ****	Saint Maarten thick-tailed gecko	2011	-	DD
**SNAKES**				
***Acanthophis cryptamydros*** *******	Kimberley death adder	2015	-	VU
***Azemiops kharini ****	White-headed fea viper	2013	-	NE
***Ophryacus smaragdinus*** *******	Emerald horned pitviper	2015	-	NE
**AMPHIBIANS**				
***Cruziohyla sylviae*** ********	Sylvia’s tree frog	2018	-	NE
***Rhacophorus helenae ****	Helen’s tree frog	2012	-	EN
***Theloderma auratum*** ********	Golden bug-eyed frog	2018	-	NE
***Theloderma lateriticum *****	Brick-red bug-eyed Frog	2009	-	LC
***Theloderma nebulosum*** ********	Misty moss frog	2011	-	EN
***Theloderma palliatum ****	Cloaked moss frog	2011	-	EN
***Theloderma vietnamense ****	South-Vietnamese bug-eyed frog	2015	-	LC
***Tylototriton shanorum ****	Taunggyi Crocodile Newt	2014	II (2019)	VU

^1^ (IUCN Red List Classification in brackets) = so far only recommended. ^2^ IUCN Red List Classification changed since our first study period.

## References

[1] IPBES (2019). Summary for Policymakers of the Global Assessment Report on Biodiversity and Ecosystem Services of the Intergovernmental Science-Policy Platform on Biodiversity and Ecosystem Services.

[2] Scheffers B., Oliveira B., Lamb I., Edwards D. (2019). Global wildlife trade across the tree of life. Science.

[3] Marshall B., Strine C., Hughes A. (2020). Thousands of reptile species threatened by under-regulated global trade. Nat. Commun..

[4] Auliya M., Altherr S., Ariano-Sanchez D., Baard E.H., Brown C., Brown R.M., Cantu J.-C., Gentile G., Gildenhuys P., Henningheim E. (2016). Trade in live reptiles, its impact on wild populations, and the role of the European market. Biol. Conserv..

[5] Auliya M., García-Moreno J., Schmidt B.R., Valbuena-Ureña E., Hoogmoed M.S., Fisher M.C., Pasmans F., Henle K., Bickford D., Martel A. (2016). The global amphibian trade flows through Europe: The need for enforcing and improving legislation. Biodivers. Conserv..

[6] Rowley J.J., Shepherd C.R., Stuart B.L., Nguyen T.Q., Hoang H.D., Cutajar T.P., Wogan G.O.U., Phimmachak S. (2016). Estimating the global trade in Southeast Asian newts. Biol. Conserv..

[7] Bush E.R., Baker S.E., Macdonald D.W. (2014). Global trade in exotic pets 2006–2012. Conserv. Biol..

[8] Ngo H.N., Nguyen T.Q., Phan T.Q., Van Schingen M., Ziegler T. (2019). A case study on trade in threatened Tiger Geckos (*Goniurosaurus*) in Vietnam including updated information on the abundance of the Endangered *G. catbaensis*. Nat. Conserv..

[9] Yang J.-H., Chan B.P.-L. (2015). Two new species of the genus *Goniurosaurus* (Squamata: Sauria: Eublepharidae) from southern China. Zootaxa.

[10] Lyons J.A., Natusch D.J.D. (2013). Effects of consumer preferences for rarity on the harvest of wild populations within a species. Ecol. Econ..

[11] Stuart B., Rhodin A., Grismer L., Hansel T. (2006). Scientific description can imperil species. Science.

[12] Janssen J., Shepherd C. (2018). Challenges in documenting trade in non CITES-listed species: A case study on crocodile skinks (*Tribolonotus* spp.). J. Asia-Pac. Biodivers..

[13] Challender D.W., Hoffmann M., Hoffmann R., Scott J., Robinson J.E., Cremona P., Hilton-Taylor C., Jenkins R.K.B., Malsch K., Conde D. (2019). Criteria for CITES species protection. Science.

[14] Frank E., Wilcove D. (2019). Long delays in banning trade in threatened species-Scientific knowledge should be applied with more urgency. Science.

[15] Altherr S., Lameter K. (2020). Stolen Wildlife III—The EU Is a Main Hub and Destination for Illegally Caught Exotic Pets.

[16] Janssen J., de Silva A. (2019). The presence of protected reptiles from Sri Lanka in international commercial trade. TRAFFIC Bull..

[17] Altherr S. (2014). Stolen Wildlife–Why the EU Needs to Tackle Smuggling of Nationally Protected Species.

[18] Flecks M., Weinsheimer F., Böhme W., Chenga J., Lötters S., Rödder D. (2012). Watching extinction happen: The dramatic population decline of the critically endangered Tanzanian turquoise dwarf gecko, *Lygodactylus williamsi*. Salamandra.

[19] Altherr S., Lameter K., Cantu J.C. (2019). Trade in nationally protected lizards from Australia, Cuba, and Mexico—and the EU’s role as a main destination. TRAFFIC Bull..

[20] Stoner S., Nijman V. (2015). The case for CITES Appendix I-listing of Earless Monitor Lizards *Lanthanotus borneensis*. TRAFFIC Bull..

[21] Janssen J., Leupen B. (2019). Traded under the radar: Poor documentation of trade in nationally-protected non-CITES species can cause fraudulent trade to go undetected. Biodivers. Conserv..

[22] Puritz A., Weller C. (2018). Illegal Wildlife Trade and the EU: Legal Approaches. Legal Analysis, Commissioned by Pro Wildlife.

[23] Altherr S., Schuller A., Fischer A. (2016). Stolen Wildlife II–Why the EU Still Needs to Tackle Smuggling of Nationally Protected Species.

[24] Musing L., Norwisz M., Kloda J., Kecse-Nagy K., TRAFFIC, WWF (2018). Wildlife Trade in Belgium: An Analysis of CITES Trade and Seizure Data.

[25] Noseworthy J. (2017). Cold-Blooded Conflict: Tackling the Illegal Trade in Endemic Caribbean Island Reptiles. Master’s Thesis.

[26] Robinson J., Griffiths R., Fraser I.M., Raharimalala J., Roberts D., St. John F. (2015). Dynamics of the global trade in live reptiles: Shifting trends in production and consequences for sustainability. Biol. Conserv..

[27] Schneeweiss N., Hintzmann J., Lippert J., Stein M., Thiesmeier B. (2014). Amphibien und Reptilienhandel als Gefährdungsfaktor für heimische Populationen. Z. für Feldherpetologie.

[28] Auliya M. (2003). Hot Trade in Cool Creatures: A Review of the Live Reptile Trade in the European Union in the 1990s with a Focus on Germany.

[29] Shepherd C., Janssen J., Noseworthy J. (2019). A case for listing the Union Island gecko *Gonatodes daudini* in the Appendices of CITES. Glob. Ecol. Conserv..

[30] EUROSTAT (2018). Import Data for Live Reptiles to EU MEMBER States 2008–2017.

[31] EU Commission (2016). EU Action Plan Against Wildlife Trafficking.

[32] Altherr S., Freyer D., Lameter K. (2020). Strategien zur Reduktion der Nachfrage nach als Heimtiere Gehaltenen Reptilien, Amphibien und kleinen Säugetieren.

[33] The Reptile Database. http://www.reptile-database.org/.

[34] AmphibiaWeb (2020). Database.

[35] NMNH (2018). Division of Mammals Collections. National Museum of Natural History.

[36] IUCN (2020). The IUCN RED List of Threatened Species. http://www.iucnredlist.org/.

[37] Wissenschaftliches Informationssystem zum Internationalen Artenschutz, WISIA. https://www.wisia.de/.

[38] Gray A. (2018). Review of the genus *Cruziohyla* (Anura: Phyllomedusidae), with description of a new species. Zootaxa.

[39] Addelman M. (2018). Spectacular Frog Identified as New Species. https://phys.org/news/2018-07-spectacular-frog-species.html.

[40] Poyarkov N., Kropachev I., Gogoleva S., Orlov N. (2018). A new species of the genus *Theloderma* Tschudi, 1838 (Amphibia: Anura: Rhacophoridae) from Tay Nguyen Plateau, central Vietnam. Zool. Res..

[41] Faizi H., Rastegar-Pouyani N., Rastegar-Pouyani E., Nazarov R., Heidari N., Zangi B., Orlova V., Poyarkov N. (2017). A new species of *Eumeces* Wiegmann 1834 (Sauria: Scincidae) from Iran. Zootaxa.

[42] Meiri S. (2016). Small, rare and trendy: Traits and biogeography of lizards described in the 21st century. J. Zool..

[43] Meiri S., Bauer A.M., Allison A., Castro-Herrera F., Chirio L., Colli G., Das I., Doan T.M., Glaw F., Grismer L.L. (2018). Extinct, obscure or imaginary: The lizard species with the smallest ranges. Divers. Distrib..

[44] Gifford M., Powell R. (2019). *Leiocephalus sixtoi*. Catalogue of American Amphibians and Reptiles (CAAR) 923.

[45] Köhler G., Bobadilla M., Hedges S. (2016). A new dune-dwelling lizard of the genus *Leiocephalus* (Iguania, Leiocephalidae) from the Dominican Republic. Zootaxa.

[46] Grünwald C., Jones J., Franz-Chávez H., Ahumada-Carrillo I. (2015). A new species of *Ophryacus* (Serpentes: Viperidae: Crotalinae) from eastern Mexico, with comments on the taxonomy of related pitvipers. Mesoam. Herpet..

[47] Ramírez-Bautista A., Hernández-Salinas U., Cruz-Elizalde R., Berriozabal-Islas C., Moreno-Lara I., DeSantis D.L., Johnson J., García-Padilla E., Mata-Silva V., Wilson L.D. (2020). The herpetofauna of Hidalgo, Mexico: Composition, distribution, and conservation status. Amphib. Reptile Conserv..

[48] Maddock S., Ellis R., Doughty P., Smith L., Wüster W. (2015). A new species of death adder (*Acanthophis*: Serpentes: Elapidae) from north-western Australia. Zootaxa.

[49] Cogger H., Ellis R., Zichy-Woinarski J., Oliver P., Shea G. (2017). *Acanthophis cryptamydros*. The IUCN Red List of Threatened Species.

[50] Batsakis A. New Snake Is among the World’s Most Venomous. Article in *Australian Geographic*, 2 October 2015. https://www.australiangeographic.com.au/news/2015/10/meet-the-kimberley-death-adder/.

[51] Amarasinghe A., Karunarathna D., Hallermann J., Fujinuma J., Grillitsch H., Campbell P. (2014). A new species of the genus *Calotes* (Squamata: Agamidae) from high elevations of the Knuckles massif of Sri Lanka. Zootaxa.

[52] Gibson C., de Silva A., Tognelli M.F., Karunarathna S. (2020). Assess to Plan: Conservation Action Planning for the Snakes and Lizards of Sri Lanka.

[53] (2019). CITES CoP18 Prop. 23. Proposal to include *Calotes nigrilabris* and *Calotes pethiyagodai* in Appendix I. https://cites.org/sites/default/files/eng/cop/18/prop/010319/E-CoP18-Prop-23.pdf.

[54] IUCN SSC Amphibian Specialist Group (2016). *Theloderma nebulosum*.  Technical Report for The IUCN Red List of Threatened Species.

[55] Rowley J., Le D., Hoang H., Dau V., Cao T. (2011). Two new species of *Theloderma* (Anura: Rhacophoridae) from Vietnam. Zootaxa.

[56] Panitvong N., Sumontha M., Konlek K., Kunya K. (2010). *Gekko lauhachindai* sp. nov., a new cave-dwelling gecko (Reptilia: Gekkonidae) from central Thailand. Zootaxa.

[57] Sumontha M., Cota M. *Gekko lauhachindai*. Technical Report for The IUCN Red List of Threatened Species 2018. https://www.researchgate.net/publication/330810861_Gekko_lauhachindai_THE_IUCN_RED_LIST_OF_THREATENED_SPECIES_2018.

[58] Whitaker A., Sadlier R. *Eurydactylodes occidentalis*. Technical Report for The IUCN Red List of Threatened Species 2011.

[59] Bauer A., Jackman T., Sadlier R., Whitaker A., Grandcolas P. (2009). Review and phylogeny of the New Caledonian diplodactylid gekkotan genus *Eurydactylodes* Wermuth, 1965, with the description of a new species. Zoologia Neocaledonica 7. Biodiversity Studies in New Caledonia.

[60] Jensen T., Auliya M., Burgess N., Aust P., Pertoldi C., Strand J. (2019). Exploring the international trade in African snakes not listed on CITES: Highlighting the role of the internet and social media. Biodivers. Conserv..

[61] Sigouin A., Pinedo-Vasquez M., Nasi R., Poole C., Horne B., Lee T. (2017). Priorities for the trade of less charismatic freshwater turtle and tortoise species. J. Appl. Ecol..

[62] Nijman V., Shepherd C., Sanders K. (2012). Over-exploitation and illegal trade of reptiles in Indonesia. Herp. J..

[63] Todd B., Willson J., Gibbons J., Sparling D.W., Linder G., Bishop C.A., Krest S. (2010). The Global Status of Reptiles and Causes of Their Decline. Ecotoxicology of Amphibians and Reptiles.

[64] Yaap B., Paoli G., Angki A., Wells P., Wahyudi D., Auliya M. (2012). First record of the Borneo Earless Monitor *Lanthanotus borneensis* (Steindachner, 1877) (Reptilia: Lanthanotidae) in West Kalimantan (Indonesian Borneo). J. Threat. Taxa.

[65] Holden M., McDonald-Madden E. (2017). High prices for rare species can drive large populations extinct: The anthropogenic Allee effect revisited. J. Theor. Biol..

[66] Angulo E., Deves A., Saint Jalmes M., Courchamp F. (2009). Fatal attraction: Rare species in the spotlight. Proc. R. Soc. B..

[67] Brook B., Sodhi N. (2006). Rarity bites. Nature.

[68] Hall R., Milner-Gulland E., Courchamp F. (2008). Endangering the endangered: The effects of perceived rarity on species exploitation. Conserv. Lett..

[69] Sung Y., Fong J. (2018). Assessing consumer trends and illegal activity by monitoring the online wildlife trade. Biol. Conserv..

[70] Lindenmayer D., Scheele B. (2017). Do not publish: Limiting open-access information on rare and endangered species will help to protect them. Science.

[71] de Fraga R., Torralvo K. (2019). New record of the fringed leaf frog, *Cruziohyla craspedopus* (Anura: Phyllomedusidae) extends its eastern range limit. Acta Amazon..

[72] Moraes L., Pavan D. (2017). Another puzzle piece: New record of the fringed leaf frog, *Cruziohyla craspedopus* (Funkhouser, 1957) (Anura: Phyllomedusidae), in the eastern Amazon Rainforest. Check List.

[73] Menegon M., Davenport T., Howell K. (2011). Description of a new and critically endangered species of *Atheris* (Serpentes: Viperidae) from the Southern Highlands of Tanzania, with an overview of the country’s tree viper fauna. Zootaxa.

[74] Eskew E.A., Ross N., Zambrana-Torrelio C., Karesh W. (2019). The CITES Trade Database is not a “global snapshot” of legal wildlife trade: Response to Can et al., 2019. Glob. Ecol. Conserv..

[75] Alacs E., Georges A. (2008). Wildlife across our borders: A review of the illegal trade in Australia. Aust. J. Forensic Sci..

[76] UNODC (2020). World Wildlife Crime Report 2020—Trafficking in Protected Species.

[77] Scanlon J. Time to End the Scourge of Wildlife Crime. Speech at Special Event in the House of Lords on 3 March, UN World Wildlife Day. https://www.independent.co.uk/voices/campaigns/GiantsClub/time-to-end-the-scourge-of-wildlife-crime-a9372651.html.

[78] European Parliament (2017). Resolution of 2 March 2017 on EU Common Commercial Policy in the context of wildlife sustainability imperatives. OJEU.

[79] European Parliament (2018). Resolution of 24 November 2016 on EU action plan against wildlife trafficking. OJEU.

[80] European Parliament (2016). Key objectives for the CITES CoP 17 meeting in Johannesburg. OJEU.

